# Cardiac Sarcoidosis: A Literature Review of Current Recommendations on Diagnosis and Management

**DOI:** 10.7759/cureus.41451

**Published:** 2023-07-06

**Authors:** Rutul Patel, Anuja Mahesh Mistry, Venkatachalam Mulukutla, Krupal Prajapati

**Affiliations:** 1 Internal Medicine, Texas Tech University Health Sciences Center, El Paso, USA; 2 Interventional Cardiology, The Hospitals of Providence, El Paso, USA; 3 Internal Medicine, Nathiba Hargovandas Lakhmichand (NHL) Municipal Medical College, Ahmedabad, IND

**Keywords:** non caseating granuloma, atrio-ventricular block, implantable cardiac defibrillator (icd), late gadolinium enhacement, mri cardiac, anti-inflammatory agents, immunosuppressive therapy, cardiac sarcoidosis

## Abstract

Cardiac sarcoidosis (CS) is a rare multisystem disorder characterized by granulomatous infiltration of the myocardium, which can lead to significant morbidity and mortality. Its clinical manifestations range from asymptomatic conduction abnormalities to severe heart failure (HF) and sudden cardiac death. This comprehensive review aims to provide an overview of the diagnosis, clinical features, and current medical management strategies for CS. Additionally, the role of implantable cardioverter-defibrillators (ICDs) and the potential use of positron emission tomography in guiding management decisions are explored. A comprehensive understanding of the medical management of CS is essential for improving patient outcomes and guiding future research endeavors.

## Introduction and background

Cardiac sarcoidosis (CS) is a rare but potentially life-threatening condition characterized by the infiltration of noncaseating granulomas in the myocardium. The disease can lead to various clinical manifestations, including arrhythmias, heart failure (HF), and conduction abnormalities. Early and accurate diagnosis is crucial for appropriate management and improved outcomes. The diagnosis of CS can be challenging due to its heterogeneous presentation. Various diagnostic approaches, including imaging modalities such as cardiac magnetic resonance (CMR) imaging, PET, and gallium-67 scintigraphy, are utilized to identify cardiac involvement [1]. CS may present with a wide range of clinical features, which can vary from asymptomatic cases to life-threatening arrhythmias or HF. Common clinical manifestations include palpitations, syncope, dyspnea, and chest pain. Arrhythmias, particularly ventricular tachycardia (VT) and atrioventricular (AV) block, are frequently observed. Corticosteroids, such as prednisone, are the cornerstone of immunosuppressive therapy in CS. They have shown efficacy in reducing cardiac inflammation and improving symptoms. Additionally, other immunosuppressive agents like methotrexate, azathioprine, and mycophenolate mofetil are used as steroid-sparing agents or in refractory cases [2]. HF management in CS involves standard therapies such as diuretics, beta-blockers, and angiotensin-converting enzyme inhibitors (ACE inhibitors) or angiotensin receptor blockers (ARBs). Device therapy, including implantable cardioverter-defibrillators (ICDs) and cardiac resynchronization therapy, may be indicated for patients at risk of life-threatening arrhythmias or with significant HF [3]. By synthesizing the existing evidence, we aim to guide clinicians in making informed decisions regarding the diagnosis and medical management of this challenging condition.

## Review

Epidemiology

There are notable differences in the incidence and prevalence of sarcoidosis among different ethnicities. Based on comprehensive nationwide register data from Sweden, the incidence of sarcoidosis was found to be 11.5 per 100,000 individuals [4], slightly surpassing the estimated rates in the United States (approximately 8-11 per 100,000) [5]. African American women seem to have the highest incidence and prevalence [6]. However, the exact reasons for these variations remain unclear and require further investigation. About 5% of sarcoidosis patients have clinical manifestations of CS, while around 25% of patients have a clinically silent disease that gets detected with imaging and autopsy [7]. One of the significant concerns in CS is the development of HF. It has been reported that approximately 20-30% of patients with CS may develop HF during the course of their disease. The risk of HF in these patients underscores the importance of early detection, accurate diagnosis, and appropriate management strategies to improve outcomes. It is worth noting that the epidemiology of CS is still not well-defined, and there are challenges in estimating the true prevalence and incidence due to potential underdiagnosis and variations in diagnostic approaches. Further research and larger population-based studies are needed to better understand the epidemiology and risk factors associated with CS.

Pathogenesis

CS involves a complex interplay of factors contributing to its pathogenesis, including genetic susceptibility, immune dysregulation, possibly microbial agents, and environmental exposures. In sarcoidosis, the formation of granulomas is hypothesized to occur as a result of an exaggerated immune response to an unknown antigen in individuals with a genetic predisposition. Certain human leukocyte antigen (HLA) alleles, such as HLA-DR17, DRB1*03, HLA-DR15, and HLA-DR14, have been associated with distinct disease courses [8]. The pathogenesis of CS involves an exaggerated TH1 cell response, producing various cytokines such as interleukin (IL)-2, interferon γ, and IL-12. Subsequently, the fibrotic transformation of chronic granulomas involves a switch to TH2 responses, accompanied by factors like platelet-derived growth factor-B, insulin-like growth factor 1 [9].

The hallmark histopathological finding of sarcoidosis is characterized by the presence of well-formed, discrete noncaseating granulomas. These granulomas consist of epithelioid histiocytes and multi-nucleated giant cells, surrounded by sparse lymphocytes, plasma cells, and fibroblasts at the periphery. The granuloma of sarcoidosis is sometimes also known as naked granuloma due to sparse lymphocytes. These extensive granulomas can lead to the destruction of normal tissue architecture. In CS, the granulomas most frequently involve the myocardium and are usually located in the basal inter-ventricular septum, left ventricular (LV) free wall, right ventricle, and atria [10]. CS-related granulomatous inflammation triggers a repair response with scarring which is patchy, unlike confluent subendocardial or transmural involvement seen in myocardial infarction [11]. Granulomas in the inter-ventricular septum can cause conduction blocks, while patchy myocardial scarring creates a substrate for ventricular tachyarrhythmias, focal aneurysms, and systolic or diastolic LV dysfunction.

Clinical manifestations

Clinical features of CS vary from palpitations, syncope, orthopnea, dyspnea, and peripheral edema to sudden cardiac death, depending upon the location of the granuloma. Involvement of the conduction pathways (AV node, bundle of His, and intraventricular pathways) can lead to heart block and arrhythmias. The most common clinical manifestation is an AV block, which can progress to a complete AV block and clinically present as syncope or sudden cardiac death [12]. Inflammation with secondary scarring and fibrosis can lead to anomalous reentry pathways causing ventricular tachyarrhythmias, which are associated with mortality and require ICD placement [13].

HF is another frequent manifestation of CS. HF symptoms encompass fluid retention-related manifestations, including edema, cough, and dyspnea, arising from excessive fluid accumulation, as well as symptoms associated with reduced cardiac output, such as fatigue and exercise intolerance due to inadequate blood supply to meet the body's demands. Cardiomyopathy in CS can manifest as LV systolic dysfunction, HF with preserved biventricular systolic function, or predominant right ventricular (RV) systolic dysfunction [14]. Granulomatous inflammation and fibrosis can lead to reduced ventricular compliance, restrictive cardiomyopathy and diastolic dysfunction, and the development of HF with preserved ejection fraction. RV pathology can result from direct granulomatous involvement or the burden imposed by long-standing pulmonary sarcoidosis and/or LV failure. Atrial involvement leads to supraventricular tachyarrhythmias like atrial fibrillation, atrial tachycardia, and atrial flutter. Pericardial effusion and pericarditis are manifestations of pericardial involvement.

Diagnosis 

The diagnosis of CS remains a challenge due to the absence of standard diagnosis criteria and the fact that it relies on a high index of suspicion. The combination of clinical assessment, laboratory investigations, EKG, echocardiography, CMR imaging, radionuclide scanning, PET imaging, and endomyocardial biopsy (EMB) plays a crucial role in establishing a definitive diagnosis and guiding management decisions.

Laboratory investigations serve as an initial step in the diagnostic workup. Although there are no specific biomarkers for CS, certain findings can raise suspicion. Elevations in angiotensin-converting enzyme, serum calcium, C-reactive protein, and erythrocyte sedimentation rate may be observed. Additionally, cardiac enzyme levels, including troponins and brain natriuretic peptide (BNP), may be increased, indicating myocardial injury and the presence of HF. EKG is a non-invasive and readily available tool for detecting cardiac abnormalities in CS. Common EKG findings include conduction disturbances, such as AV blocks, bundle branch blocks, and ventricular arrhythmias [15]. These findings are suggestive but not specific for CS, as they can also be seen in other cardiac conditions.

Echocardiography provides valuable information regarding cardiac structure and function. In CS, it helps to identify ventricular wall motion abnormalities, regional wall thickening, and valvular dysfunction. Reduced LV ejection fraction (LVEF) and increased wall thickness may also be observed. CMR imaging has emerged as a powerful tool in the evaluation of CS. It allows for the visualization of myocardial inflammation, granulomas, fibrosis, and regional wall motion abnormalities in a patchy distribution. Delayed gadolinium enhancement/late gadolinium enhancement (LGE) reveals multifocal myocardial lesions usually in the basal inter-ventricular septum, which is suggestive of CS. CMR imaging has high sensitivity and specificity in detecting CS-related abnormalities [16].

Radionuclide scanning using technetium-99m (Tc-99m) agents or gallium-67 (Ga-67) citrate can help to identify active inflammation in the myocardium. Increased radiotracer uptake within the heart on scintigraphy images indicates myocardial involvement. However, this method lacks specificity and cannot differentiate CS from other inflammatory or infectious processes. PET imaging with 18F-fluorodeoxyglucose (FDG) is an emerging modality for diagnosing CS. FDG-PET allows for the assessment of myocardial glucose metabolism. Increased FDG uptake in the myocardium indicates active inflammation. The combination of PET and CT imaging (PET/CT) improves accuracy by providing anatomical correlation. However, false-negative results may occur in patients with low-grade or fibrotic inflammation. The specificity of PET-CT for the diagnosis of CS is 78% with a sensitivity of 89% [17].

Hybrid PET and CMR imaging, made possible by the development of new hybrid PET-CMR systems, allows for the integration of the strengths of PET and CMR in a single acquisition. Despite the higher costs associated with these scanners, the benefits are significant, including increased accuracy and reduced risk of complications. MRI demonstrates excellent sensitivity in detecting fibrosis, while PET enables visualization and quantification of inflammatory activity and extracardiac involvement. Limited studies indicate that combining both modalities leads to improved detection rates, enhanced accuracy, and potential impact on therapeutic decision-making, providing an additional evaluation of scar and inflammation findings for comprehensive disease management, staging, and prognosis [18].

EMB involves obtaining myocardial tissue samples for histopathological analysis. The presence of noncaseating granulomas within the myocardium confirms the diagnosis of CS; thus, the test has high specificity. However, due to the patchy nature of granuloma distribution, EMB may yield false-negative results, leading to low sensitivity of 20-30% [19]. Additionally, the procedure carries a risk of complications, such as perforation and arrhythmias.

**Figure 1:**
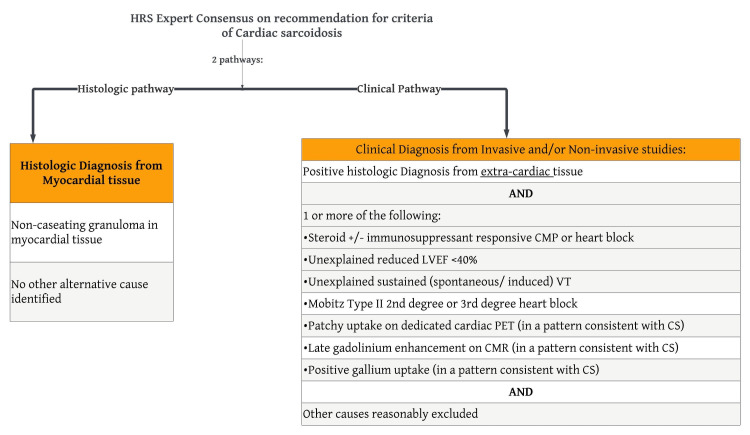
HRS expert consensus recommendations on criteria for the diagnosis of CS The criteria for the diagnosis of CS based on Heart Rhythm Society expert consensus recommendations involving 2 pathways [20]. HRS: Heart Rhythm Society; CS: cardiac sarcoidosis; CMP: cardiomyopathy; LVEF: left ventricle ejection fraction; VT: ventricular tachycardia; PET: positron emission tomography; CMR: cardiovascular magnetic resonance

**Figure 2:**
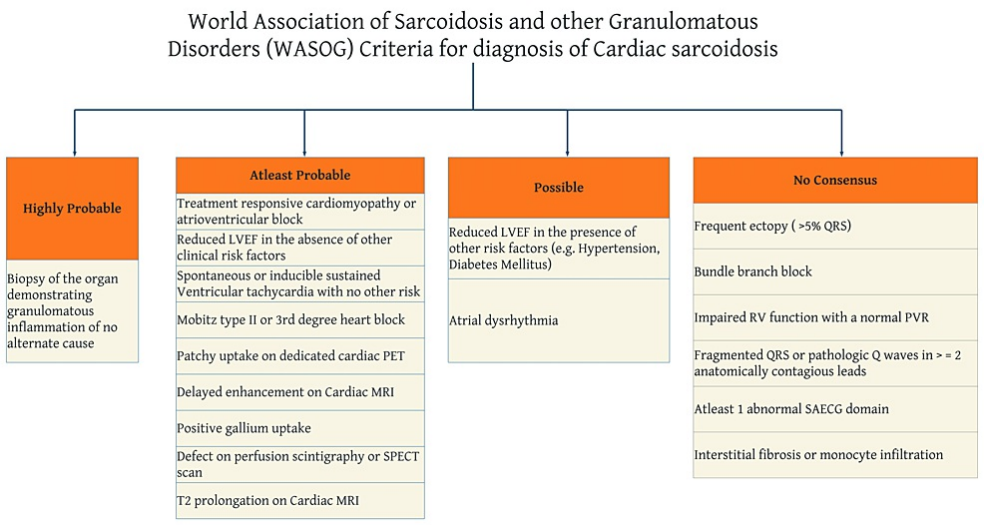
The WASOG criteria for the diagnosis of CS The WASOG criteria for the diagnosis of CS classified according to the organ assessment instrument developed by this society [21]. LVEF: left ventricle ejection fraction; PET: positron emission tomography; MRI: magnetic resonance imaging; SPECT: single-photon emission computed tomography; RV: right ventricle; PVR: pulmonary vascular resistance; SAECG: signal-averaged electrocardiogram

There are no established gold-standard criteria for the diagnosis of CS, but recommendations from the Heart Rhythm Society (HRS) (Figure 1) and the World Association of Sarcoidosis and Other Granulomatous (WASOG) Disorders (Figure 2) are summarized below.

Differential diagnosis

Distinguishing CS from other conditions can be challenging due to overlapping clinical presentations. Myocarditis, characterized by ventricular arrhythmias, HF symptoms, LGE on CMR, and abnormal uptake on FDG-PET, shares similarities with CS [22]. An EMB may be necessary to differentiate these two conditions. Arrhythmogenic RV cardiomyopathy (ARVC) presents with similar features to CS, including ventricular arrhythmias, but AV block and HF symptoms are more common in CS [23]. LGE absence in the ventricular septum and intramyocardial fat infiltration favor ARVC. Other conditions, such as prior myocardial infarction, cardiac amyloidosis, hypertrophic cardiomyopathy, Fabry disease, and hereditary hemochromatosis, can also exhibit LGE on CMR. Pattern distribution of LGE and associated clinical features aid in distinguishing these conditions from CS. Granulomas observed on EMB can also occur in tuberculosis, fungal infections, systemic vasculitis, and immunodeficiencies, but detailed clinical history, imaging findings, and specific diagnostic tests assist in ruling out these conditions [24]. Careful evaluation and a comprehensive approach are crucial to differentiate CS from these various differential diagnoses.

Management

The European Respiratory Society clinical practice guidelines on the treatment of sarcoidosis published in 2021 found the following features to be associated with increased risk for morbidity or mortality from CS [25]: age >50 years, LV ejection fraction <40%, New York Heart Association Functional Class III or IV, increased LV end-diastolic diameter, LGE on CMR imaging, VT, cardiac inflammation identified by FDG PET scan, echocardiographic evidence of abnormal global longitudinal strain, inter-ventricular septal thinning, and elevated troponin or BNP.

The management of CS requires a comprehensive and multidisciplinary approach involving cardiologists, pulmonologists, electrophysiologists, and rheumatologists to optimize patient care. This multifaceted management strategy aims to control inflammation, prevent arrhythmias, preserve ventricular function, and improve overall outcomes. In this section, we will delve deeper into the specific aspects of CS management, including immunosuppressive therapy, ICDs, and HF management.

Immunosuppressive therapy forms the cornerstone of CS management, aiming to suppress inflammation and halt disease progression. Glucocorticoids, particularly prednisone, play a central role in this treatment strategy. Steroids exert their action in the management of CS by suppressing granuloma formation. This is achieved through the inhibition of the production of interplaying cytokines and the restoration of functional CD4+ T cells while also balancing the subtypes of effector CD4+ T cells involved in the immune response [26]. Initially, high doses of prednisone, such as 0.5 mg/kg/day, are administered to achieve disease control. This intensive therapy is followed by a gradual tapering regimen over several months to a maintenance dose of 5-10 mg/day. According to retrospective analysis, it was found that prednisone doses higher than 0.5 mg/kg did not yield superior effectiveness compared to an initial dose of 0.5 mg/kg [27]. The duration of glucocorticoid therapy varies based on individual patient response and disease activity. However, efforts are made to minimize long-term glucocorticoid use due to potential adverse effects, including osteoporosis, diabetes, hypertension, and increased infection risk.

In cases where glucocorticoid monotherapy is inadequate, additional immunosuppressive agents are considered steroid-sparing agents. Methotrexate, an antimetabolite, is commonly used in combination with glucocorticoids. Administered orally at a starting dose of 5-10 mg once a week with gradual titration up to 20 mg once a week, methotrexate helps to reduce the dependence on glucocorticoids [28]. Methotrexate should be given with folic acid at a dose of 1 mg daily or 5 mg weekly. Patients on methotrexate therapy require frequent monitoring with CBC, liver function tests, and serum creatinine. Other immunosuppressants that have been used in several cases are azathioprine, mycophenolate mofetil, and leflunomide.

**Figure 3:**
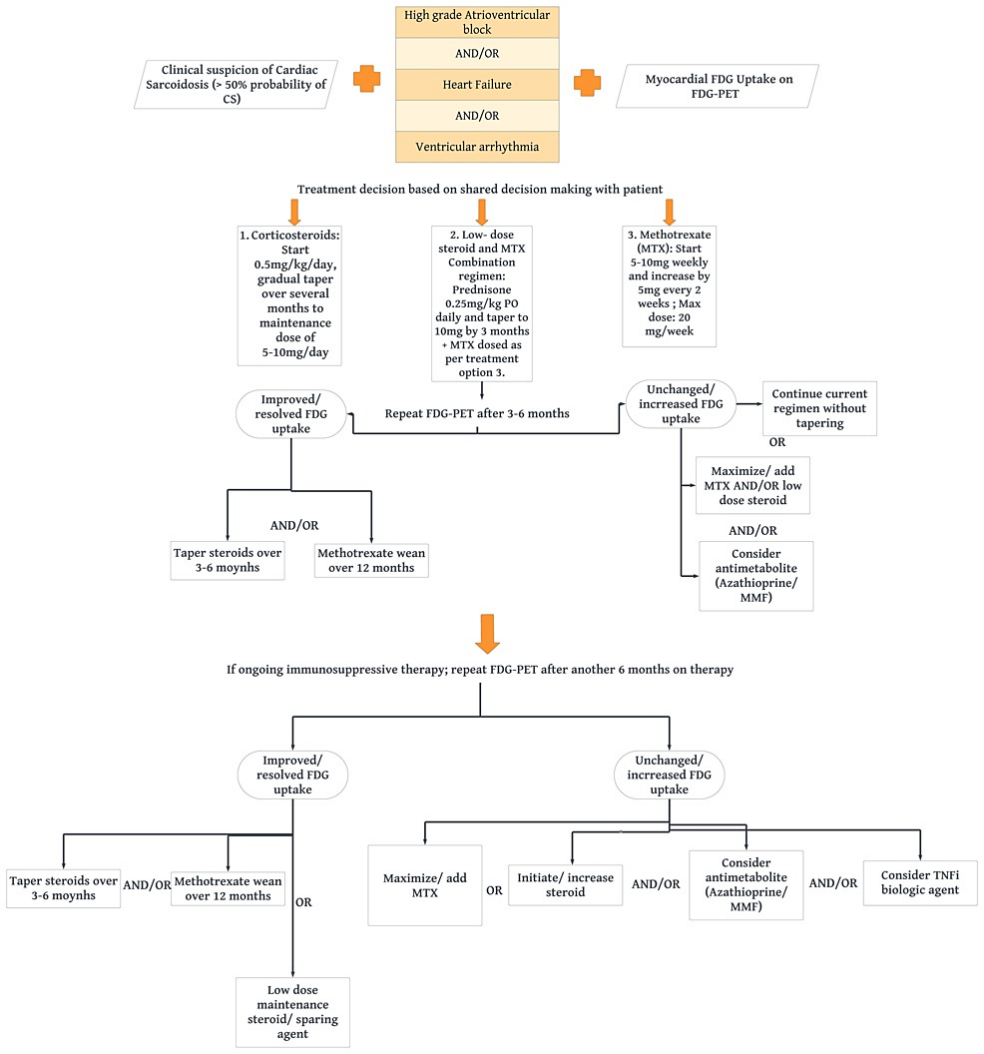
Proposed algorithm for the treatment of CS The proposed algorithm for the treatment of CS based on the article published by Gerard et al. [30]. CS: cardiac sarcoidosis; FDG: fluorodeoxyglucose; FDG-PET: fluorodeoxyglucose positron emission tomography; MTX: methotrexate; MMF: mycophenolate mofetil; TNFi: tumor necrosis factor inhibitors

Patients who do not respond to tiered therapy with corticosteroids and steroid-sparing agents, such as methotrexate, for the management of CS may require third-line treatment options. Biologic agents, specifically tumor necrosis factor-alpha (TNF-α) inhibitors, are considered in such cases. TNF-α is a pivotal cytokine involved in the formation and maintenance of granulomas, making TNF-α inhibitors, such as infliximab (a chimeric monoclonal antibody) and adalimumab (a humanized monoclonal antibody), potential treatment options for CS [29]. These biological agents work by blocking the activity of TNF-α and have been utilized in the management of CS to target the underlying inflammatory processes and reduce granuloma formation. Before initiating biological therapy, it is crucial to rule out serious active infection, conduct screening for tuberculosis, viral hepatitis, and HIV in at-risk individuals and avoid starting treatment in patients with a known or suspected malignancy. The proposed algorithm for the management of myocardial inflammation in CS is summarized below (Figure 3).

ICDs play a crucial role in managing CS-related arrhythmias and preventing sudden cardiac death. The indications for ICD implantation in CS patients include sustained VT, hemodynamically unstable VT, or a history of cardiac arrest [31]. ICDs are highly effective in terminating dangerous arrhythmias and providing life-saving interventions. The decision to implant an ICD is made based on the patient's risk profile, considering factors such as the severity of ventricular dysfunction, prior arrhythmias, and other clinical characteristics. The programming of ICDs in CS patients is tailored to detect and treat ventricular tachyarrhythmias, with higher detection rates and longer detection intervals to minimize inappropriate shocks.

Optimal HF management is also crucial in CS, as cardiac involvement can lead to impaired ventricular function and HF symptoms. ACE inhibitors or ARBs are commonly prescribed to reduce afterload and improve ventricular function. Beta-blockers, such as carvedilol or metoprolol, are used to control heart rate, reduce myocardial oxygen demand, and improve ventricular remodeling. Diuretics are utilized to manage fluid retention and alleviate symptoms of congestion. In patients with reduced ejection fraction, guideline-directed medical therapy for HF, including the use of mineralocorticoid receptor antagonists and sacubitril/valsartan, may be considered [32]. In advanced cases of CS-related HF, cardiac transplantation may be a viable option for select patients.

Regular monitoring and follow-up are essential to assess treatment response, adjust therapeutic strategies, and detect potential complications. Imaging modalities such as CMR imaging and echocardiography can provide valuable information on disease activity, ventricular function, and response to therapy. Additionally, serial measurements of serum biomarkers, including BNP and troponin levels, can aid in monitoring disease progression and guiding treatment decisions.

Ongoing research into the pathogenesis of CS and the development of targeted therapies offer promise for improved outcomes in the future. The collaborative efforts of a multidisciplinary team are paramount to providing optimal care for patients with CS, ensuring individualized treatment plans and comprehensive disease management. By combining immunosuppressive therapy, ICD implantation when indicated, and effective HF management, healthcare providers can strive to enhance the prognosis and quality of life for patients with CS.

## Conclusions

In conclusion, CS presents a complex clinical challenge, requiring a multidisciplinary approach for accurate diagnosis and optimal management. Diagnostic advancements, including CMR imaging, echocardiography, radionuclide scanning, and PET scanning, have improved disease detection and assessment. Corticosteroids remain the mainstay of treatment, targeting immune dysregulation and granuloma formation. For refractory cases, methotrexate or TNF-α inhibitors may be considered. Conduction abnormalities and arrhythmias necessitate careful monitoring and intervention, with ICD placement indicated in high-risk patients. Long-term follow-up and collaboration among healthcare professionals are crucial. Despite progress, unanswered questions and ongoing research efforts remain, offering hope for improved understanding and treatment. An individualized approach combining early diagnosis, appropriate therapy, and timely intervention is key to effectively managing CS and improving outcomes. Continued research and collaboration are essential for advancing care and addressing the complexities of CS.

## References

[1] Ueberham L, Hagendorff A, Klingel K (2023). Pathophysiological gaps, diagnostic challenges, and uncertainties in cardiac sarcoidosis. J Am Heart Assoc.

[2] Gerke AK (2020). Treatment of sarcoidosis: a multidisciplinary approach. Front Immunol.

[3] Mankad P, Mitchell B, Birnie D, Kron J (2019). Cardiac sarcoidosis. Curr Cardiol Rep.

[4] Arkema EV, Grunewald J, Kullberg S, Eklund A, Askling J (2016). Sarcoidosis incidence and prevalence: a nationwide register-based assessment in Sweden. Eur Respir J.

[5] Baughman RP, Field S, Costabel U (2016). Sarcoidosis in America. Analysis based on health care use. Ann Am Thorac Soc.

[6] Cozier YC, Berman JS, Palmer JR, Boggs DA, Serlin DM, Rosenberg L (2011). Sarcoidosis in black women in the United States: data from the Black Women's Health Study. Chest.

[7] Birnie DH, Nery PB, Ha AC, Beanlands RS (2016). Cardiac sarcoidosis. J Am Coll Cardiol.

[8] Berlin M, Fogdell-Hahn A, Olerup O, Eklund A, Grunewald J (1997). HLA-DR predicts the prognosis in Scandinavian patients with pulmonary sarcoidosis. Am J Respir Crit Care Med.

[9] Lagana SM, Parwani AV, Nichols LC (2010). Cardiac sarcoidosis: a pathology-focused review. Arch Pathol Lab Med.

[10] Tavora F, Cresswell N, Li L, Ripple M, Solomon C, Burke A (2009). Comparison of necropsy findings in patients with sarcoidosis dying suddenly from cardiac sarcoidosis versus dying suddenly from other causes. Am J Cardiol.

[11] Smedema JP, van Geuns RJ, Truter R, Mayosi BM, Crijns HJ (2017). Contrast-enhanced cardiac magnetic resonance: distinction between cardiac sarcoidosis and infarction scar. Sarcoidosis Vasc Diffuse Lung Dis.

[12] Nery PB, Beanlands RS, Nair GM (2014). Atrioventricular block as the initial manifestation of cardiac sarcoidosis in middle-aged adults. J Cardiovasc Electrophysiol.

[13] Yazaki Y, Isobe M, Hiroe M (2001). Prognostic determinants of long-term survival in Japanese patients with cardiac sarcoidosis treated with prednisone. Am J Cardiol.

[14] Gilotra NA, Griffin JM, Pavlovic N (2022). Sarcoidosis-related cardiomyopathy: current knowledge, challenges, and future perspectives state-of-the-art review. J Card Fail.

[15] Cherrett C, Lee W, Bart N, Subbiah R (2023). Management of the arrhythmic manifestations of cardiac sarcoidosis. Front Cardiovasc Med.

[16] Vita T, Okada DR, Veillet-Chowdhury M (2018). Complementary value of cardiac magnetic resonance imaging and positron emission tomography/computed tomography in the assessment of cardiac sarcoidosis. Circ Cardiovasc Imaging.

[17] Youssef G, Leung E, Mylonas I (2012). The use of 18F-FDG PET in the diagnosis of cardiac sarcoidosis: a systematic review and metaanalysis including the Ontario experience. J Nucl Med.

[18] Wicks EC, Menezes LJ, Barnes A (2018). Diagnostic accuracy and prognostic value of simultaneous hybrid 18F-fluorodeoxyglucose positron emission tomography/magnetic resonance imaging in cardiac sarcoidosis. Eur Heart J Cardiovasc Imaging.

[19] Uemura A, Morimoto S, Hiramitsu S, Kato Y, Ito T, Hishida H (1999). Histologic diagnostic rate of cardiac sarcoidosis: evaluation of endomyocardial biopsies. Am Heart J.

[20] Birnie DH, Sauer WH, Bogun F (2014). HRS expert consensus statement on the diagnosis and management of arrhythmias associated with cardiac sarcoidosis. Heart Rhythm.

[21] Judson MA, Costabel U, Drent M (2014). The WASOG sarcoidosis organ assessment instrument: an update of a previous clinical tool. Sarcoidosis Vasc Diffuse Lung Dis.

[22] Doltra A, Amundsen BH, Gebker R, Fleck E, Kelle S (2013). Emerging concepts for myocardial late gadolinium enhancement MRI. Curr Cardiol Rev.

[23] Vasaiwala SC, Finn C, Delpriore J (2009). Prospective study of cardiac sarcoid mimicking arrhythmogenic right ventricular dysplasia. J Cardiovasc Electrophysiol.

[24] Prasse A (2016). The diagnosis, differential diagnosis, and treatment of sarcoidosis. Dtsch Arztebl Int.

[25] Baughman RP, Valeyre D, Korsten P (2021). ERS clinical practice guidelines on treatment of sarcoidosis. Eur Respir J.

[26] Oswald-Richter KA, Richmond BW, Braun NA (2013). Reversal of global CD4+ subset dysfunction is associated with spontaneous clinical resolution of pulmonary sarcoidosis. J Immunol.

[27] Hiramitsu S, Morimoto S, Uemura A (2005). National survey on status of steroid therapy for cardiac sarcoidosis in Japan. Sarcoidosis Vasc Diffuse Lung Dis.

[28] Cremers JP, Drent M, Bast A (2013). Multinational evidence-based World Association of Sarcoidosis and Other Granulomatous Disorders recommendations for the use of methotrexate in sarcoidosis: integrating systematic literature research and expert opinion of sarcoidologists worldwide. Curr Opin Pulm Med.

[29] Chung ES, Packer M, Lo KH, Fasanmade AA, Willerson JT (2003). Randomized, double-blind, placebo-controlled, pilot trial of infliximab, a chimeric monoclonal antibody to tumor necrosis factor-alpha, in patients with moderate-to-severe heart failure: results of the anti-TNF Therapy Against Congestive Heart Failure (ATTACH) trial. Circulation.

[30] Giblin GT, Murphy L, Stewart GC (2021). Cardiac sarcoidosis: when and how to treat inflammation. Card Fail Rev.

[31] Birnie D, Ha AC, Gula LJ, Chakrabarti S, Beanlands RS, Nery P (2015). Cardiac sarcoidosis. Clin Chest Med.

[32] McDonagh TA, Metra M, Adamo M (2022). 2021 ESC Guidelines for the diagnosis and treatment of acute and chronic heart failure: developed by the Task Force for the diagnosis and treatment of acute and chronic heart failure of the European Society of Cardiology (ESC). With the special contribution of the Heart Failure Association (HFA) of the ESC. Eur J Heart Fail.

